# Supplementary material for: Polyoxometalate-Modified Amphiphilic Polystyrene-block-poly(2-(dimethylamino)ethyl methacrylate) Membranes for Heterogeneous Glucose to Formic Acid Methyl Ester Oxidation
Source: Nanomaterials (Basel). 2023 Sep 5;13(18):2498. doi: 10.3390/nano13182498 (PMC10536830; doi:10.3390/nano13182498)
Supplement: Supplementary file 1 [file nanomaterials-13-02498-s001.zip › nanomaterials-2519494-supplementary.pdf]

Supporting information

Polyoxometalate-Modified Amphiphilic Polystyrene-*block*-poly(2-(dimethylamino)ethyl methacrylate) Membranes for Heterogeneous Glucose to Formic Acid Methyl Ester Oxidation

Yurii Utievskyi [1], Christof Neumann [2], Julia Sindlinger [3], Konstantin Schutjajew [4], Martin Oschatz [4,5], Andrey Turchanin [2,5,6], Nico Ueberschaar [3] and Felix H. Schacher [1,5,6,*]

[1] Institute of Organic Chemistry and Macromolecular Chemistry, Friedrich Schiller University Jena, Humboldtstraße 10, 07743 Jena, Germany
[2] Institute of Physical Chemistry, Friedrich Schiller University Jena, Lessingstraße 10, 07743 Jena, Germany
[3] Mass Spectrometry Platform, Faculty of Chemistry and Earth Sciences, Friedrich Schiller University Jena, Humboldtstraße 8, 07743 Jena, Germany
[4] Institute for Technical Chemistry and Environmental Chemistry (ITUC), Friedrich Schiller University Jena, Philosophenweg 7a, 07743 Jena, Germany
[5] Center for Energy and Environmental Chemistry (CEEC), Friedrich Schiller University Jena, 07743 Jena, Germany
[6] Jena Center for Soft Matter (JCSM), Friedrich Schiller University Jena, 07743 Jena, Germany
* Correspondence: felix.schacher@uni-jena.de

Content

1. PS-*b*-PDMAEMA block copolymer characterization………………………………………………….. S3
2. BET sorption analysis plots…………………………………………………………………………………. S4
3. HPA-5 POM $H_8[PV_5Mo_7O_{40}]$ synthesis and characterization……………………………………… S5
4. PS-*b*-PDMAEMA membranes……………………………………………………………………………… S6
5. High-pressure reactor for catalytic reactions……………………………………………………………. S7
6. ICP-OES plots………………………………………………………………………………………………… S8

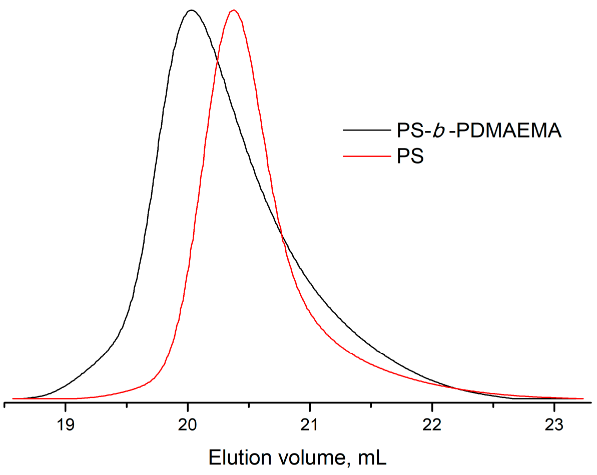

Figure S1. SEC elution traces (in THF as eluent, polystyrene calibration) of PS macroinitiator and PS-*b*-PDMAEMA block copolymer.

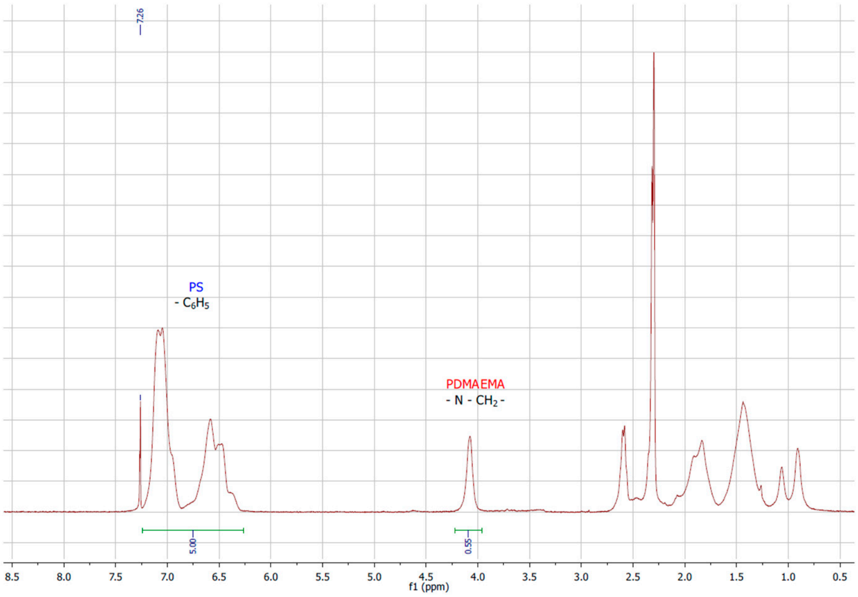

Figure S2. ^{1}H NMR spectrum of the PS-*b*-PDMAEMA block copolymer (300 MHz, $CDCl_3$).

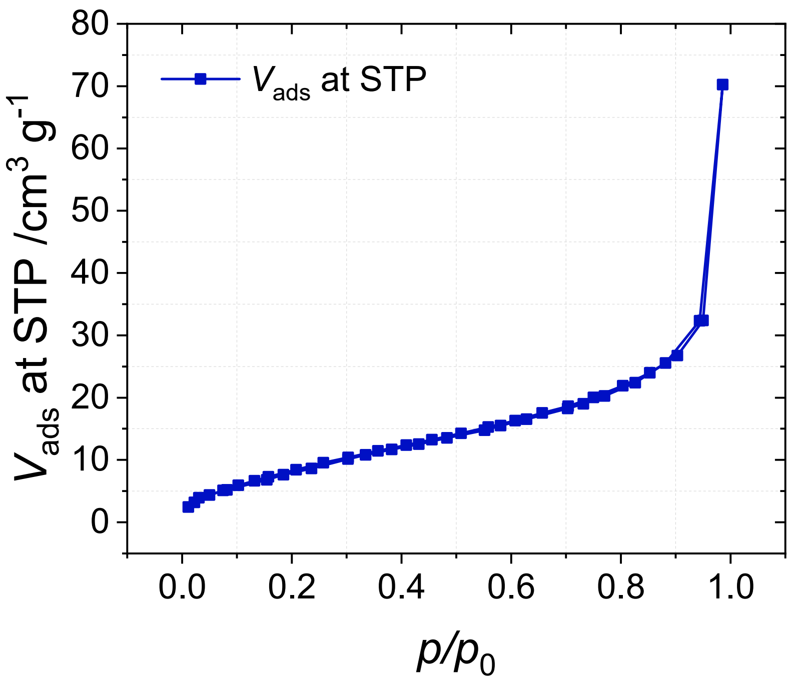

Figure S3. Nitrogen sorption isotherm of a pristine PS-*b*-PDMAEMA membrane recorded at 77 K.

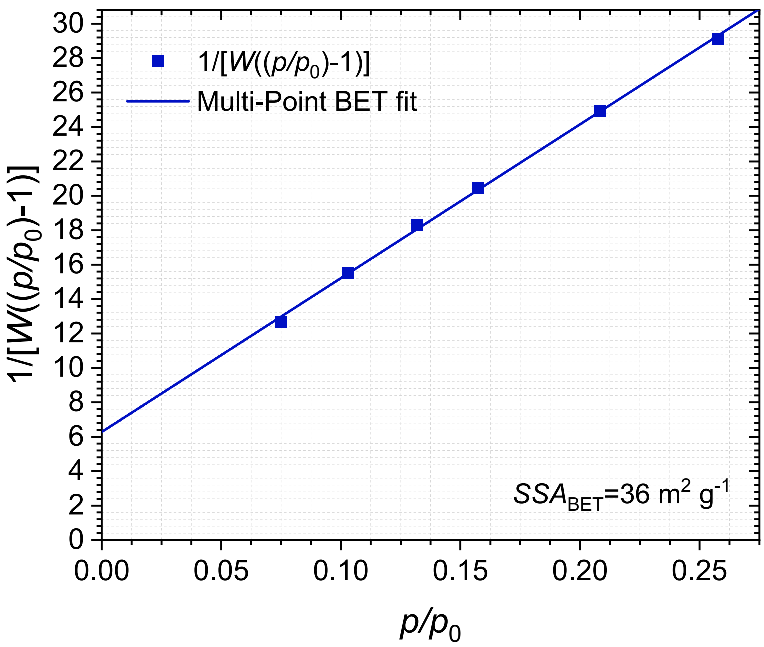

Figure S4. Multi-point BET plot of the isotherm in a p/p_0 range of 0.075-0.25.

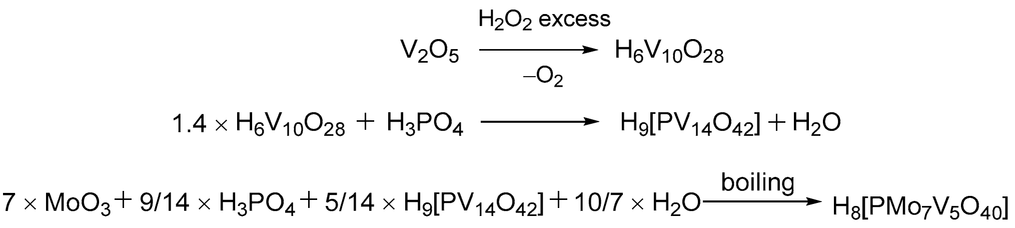

Figure S5. Three step synthesis of the HPA-5 POM $H_8[PV_5Mo_7O_{40}]$ catalyst.

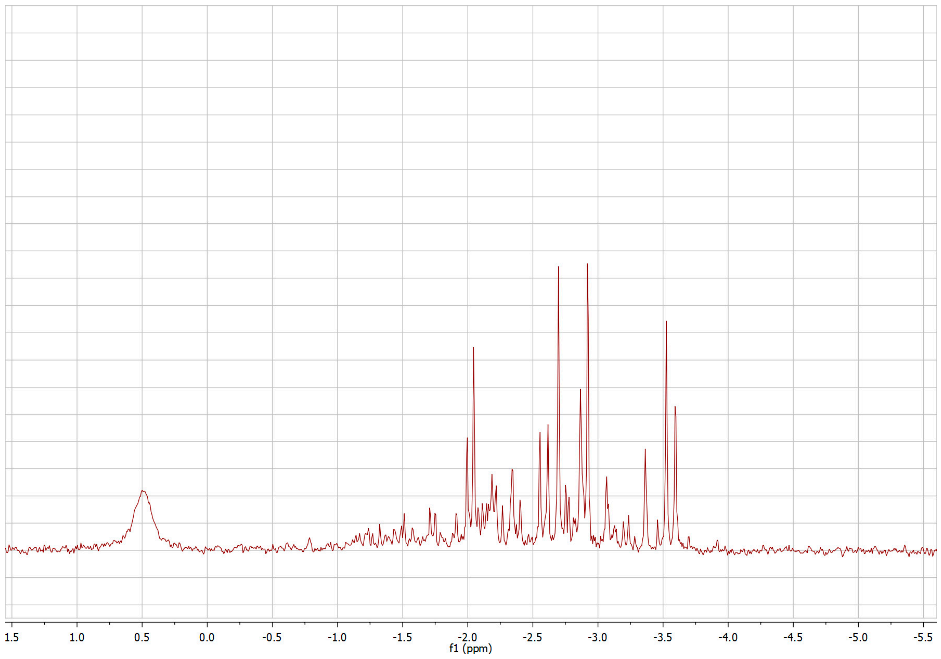

Figure S6. ^{31}P NMR spectrum of the HPA-5 POM $H_8[PV_5Mo_7O_{40}]$ catalyst (162 MHz, D_2O), showing signals of multiple positional isomers of the HPA-5 in the interval from +1.0 to -4.0 ppm, resulting from high degree of molybdenum substitution with vanadium. The peak at +0.5 ppm corresponds to the free phosphate $[PO_4]^{3-}$ anion.

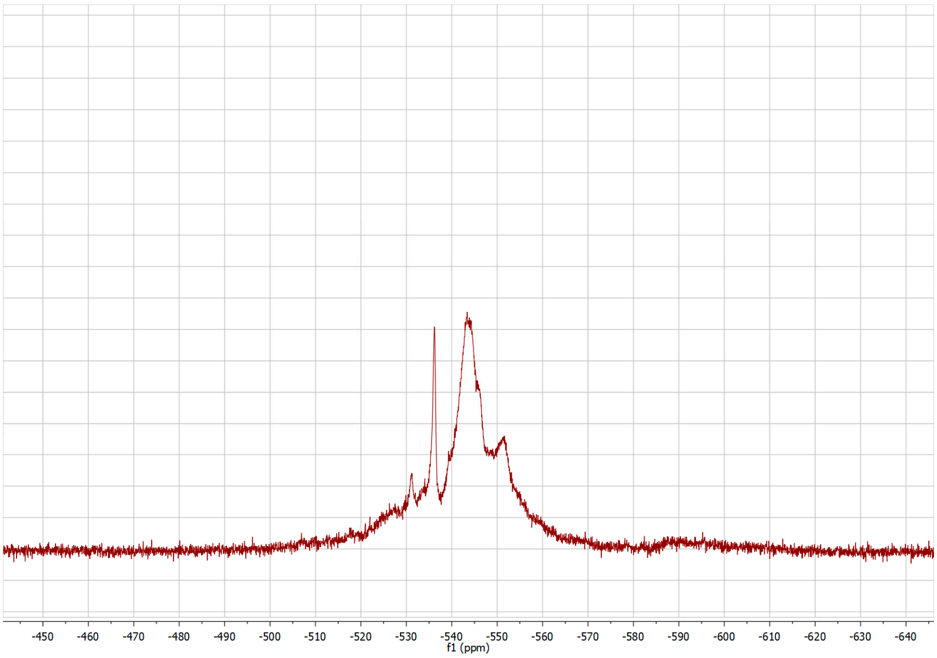

Figure S7. ^{51}V NMR spectrum of the HPA-5 POM $H_8[PV_5Mo_7O_{40}]$ catalyst showing signals of multiple positional isomers of the HPA-5 in the interval from -510 to -570 ppm, resulting from high degree of molybdenum substitution with vanadium (105 MHz, D_2O).

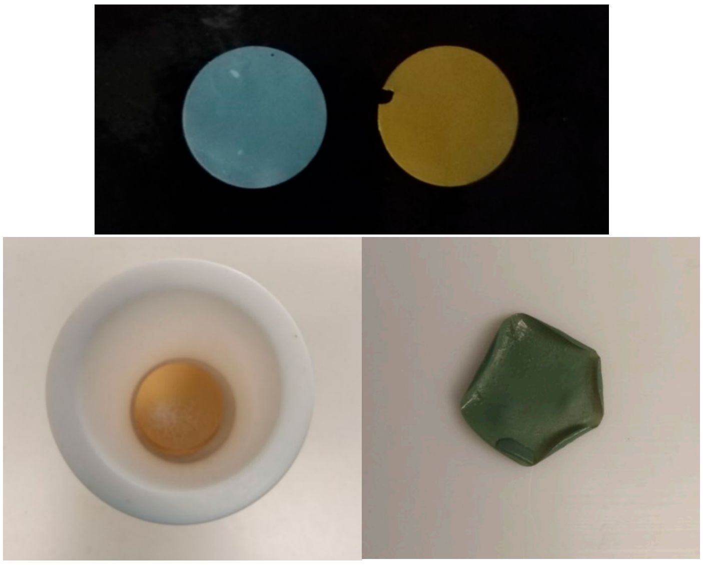

Figure S8. PS-*b*-PDMAEMA and HPA-5 POM modified catalytically active membranes (top image), catalytic membrane in the 50 mL PTFE reaction vessel (bottom-left image), catalytic membrane after five consecutive glucose oxidation cycles (bottom-right image)

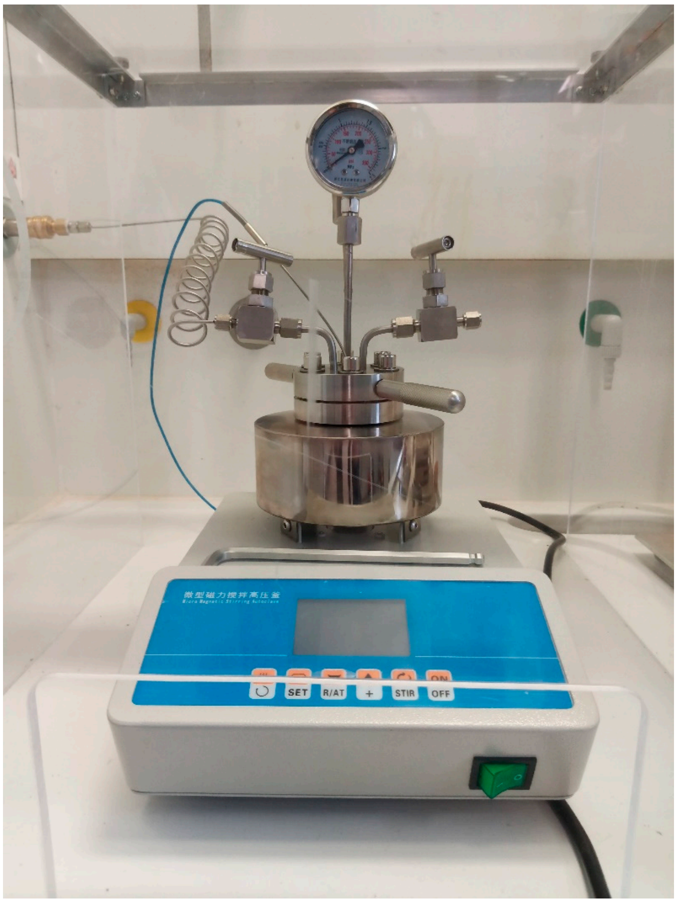

Figure S9. High-pressure OLT-HP-50 reactor connected directly to the air supply line.

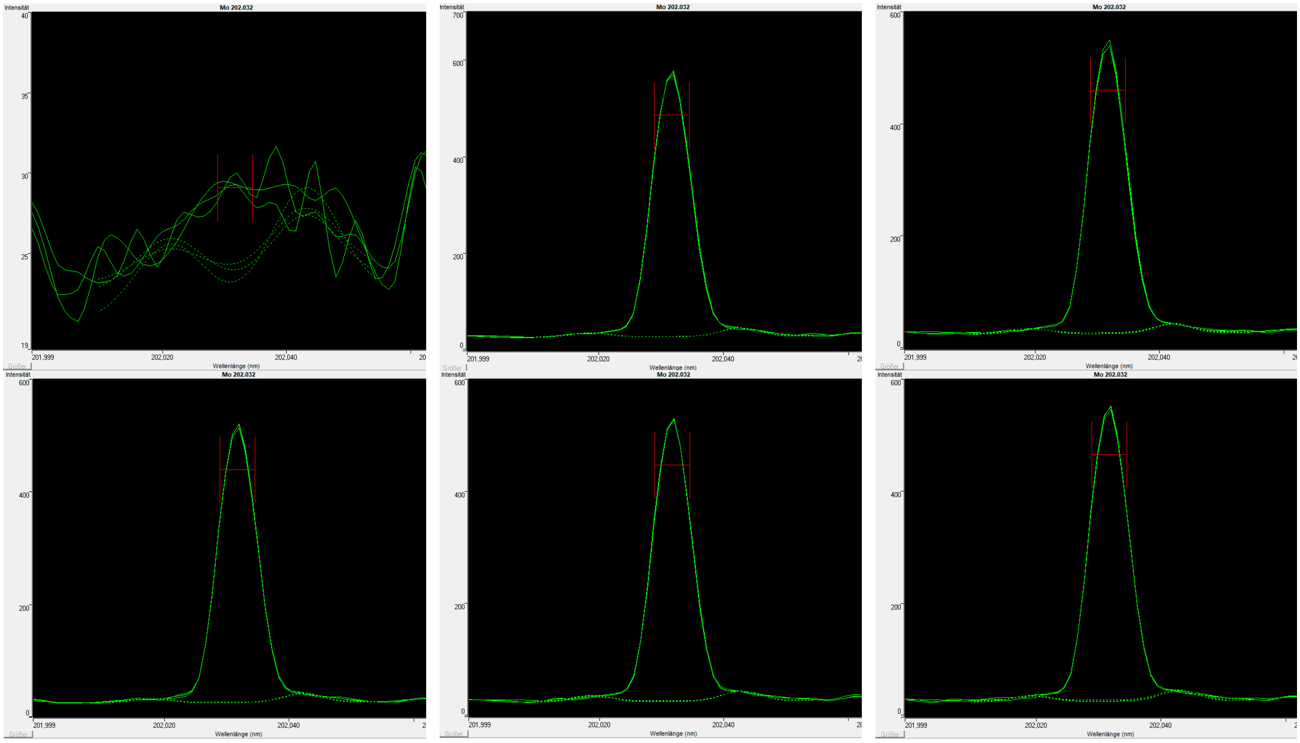

Figure S10. ICP-OES spectra of leached Mo (202.032 nm) in the reaction solutions of the catalytic set 2. Blank sample and reaction solutions from cycles 1-5 are presented from left to right.

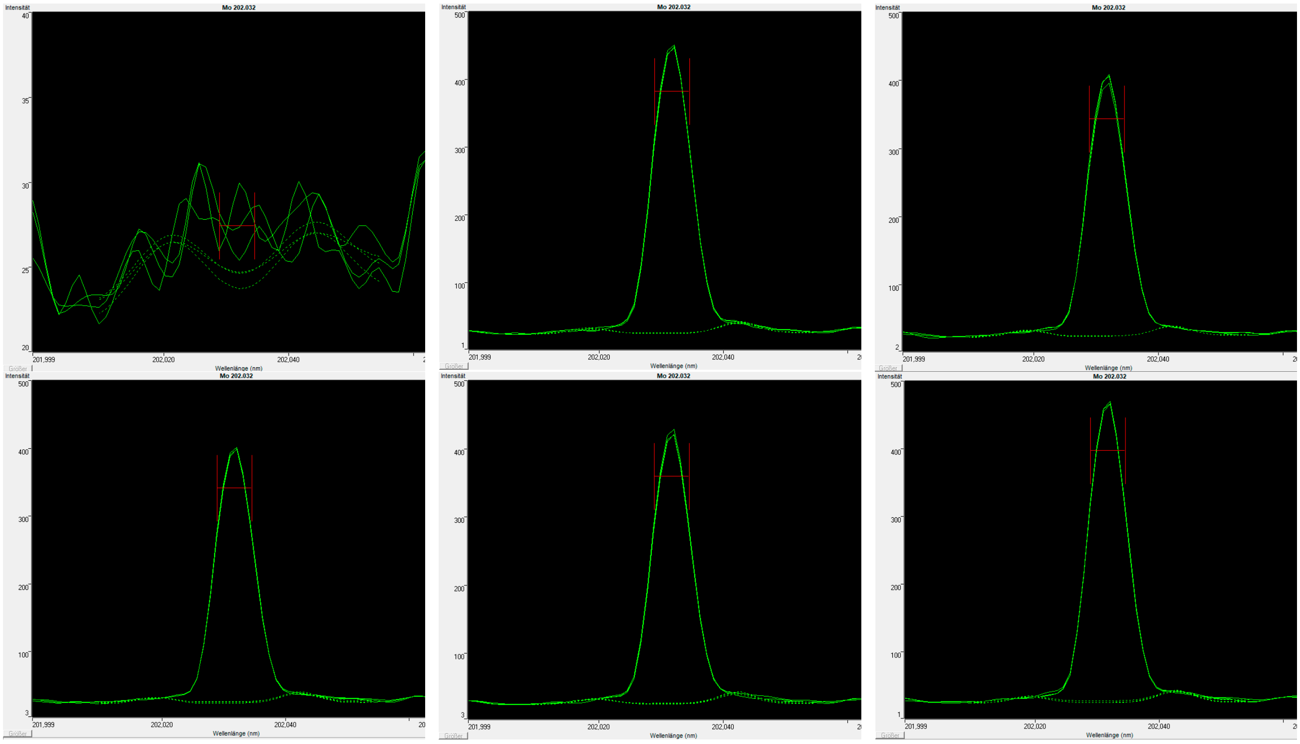

Figure S11. ICP-OES spectra of leached Mo (202.032 nm) in the reaction solutions of the catalytic set 3. Blank sample and reaction solutions from cycles 1-5 are presented from left to right.

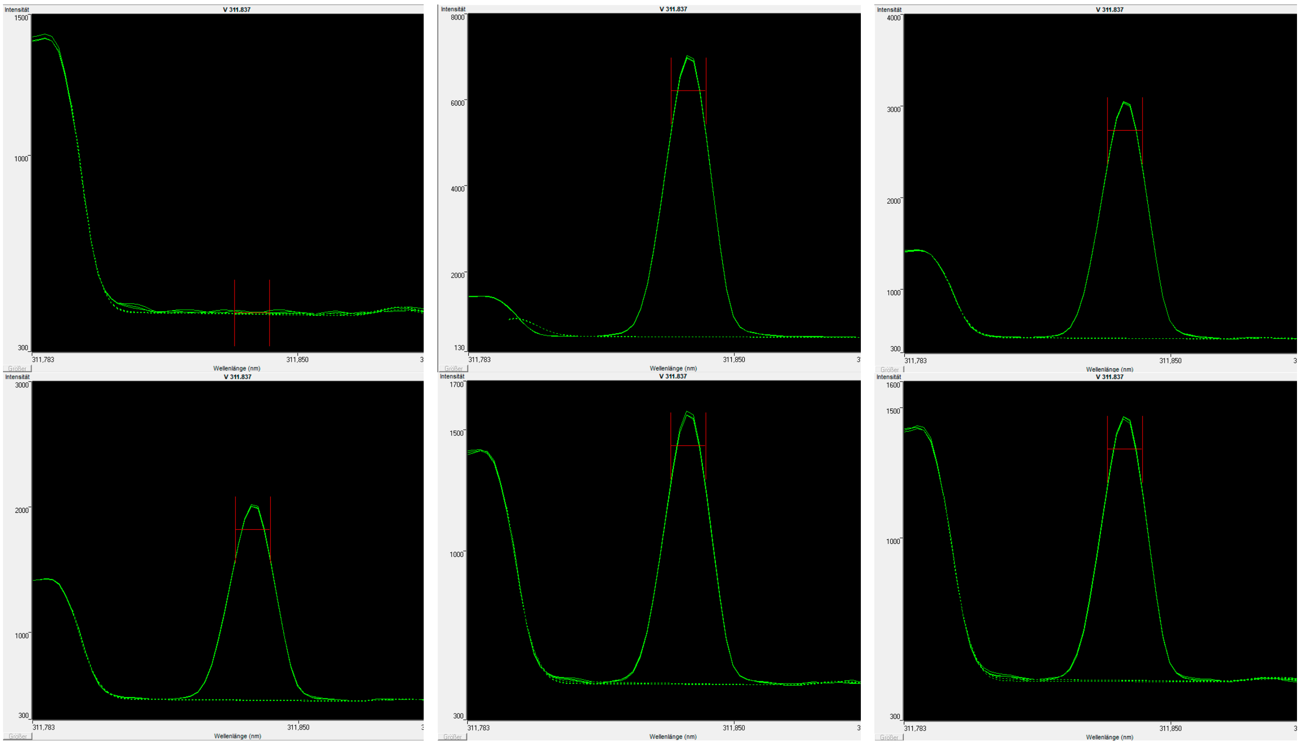

Figure S12. ICP-OES spectra of leached V (311.837 nm) in the reaction solutions of the catalytic set 2. Blank sample and reaction solutions from cycles 1-5 are presented from left to right.

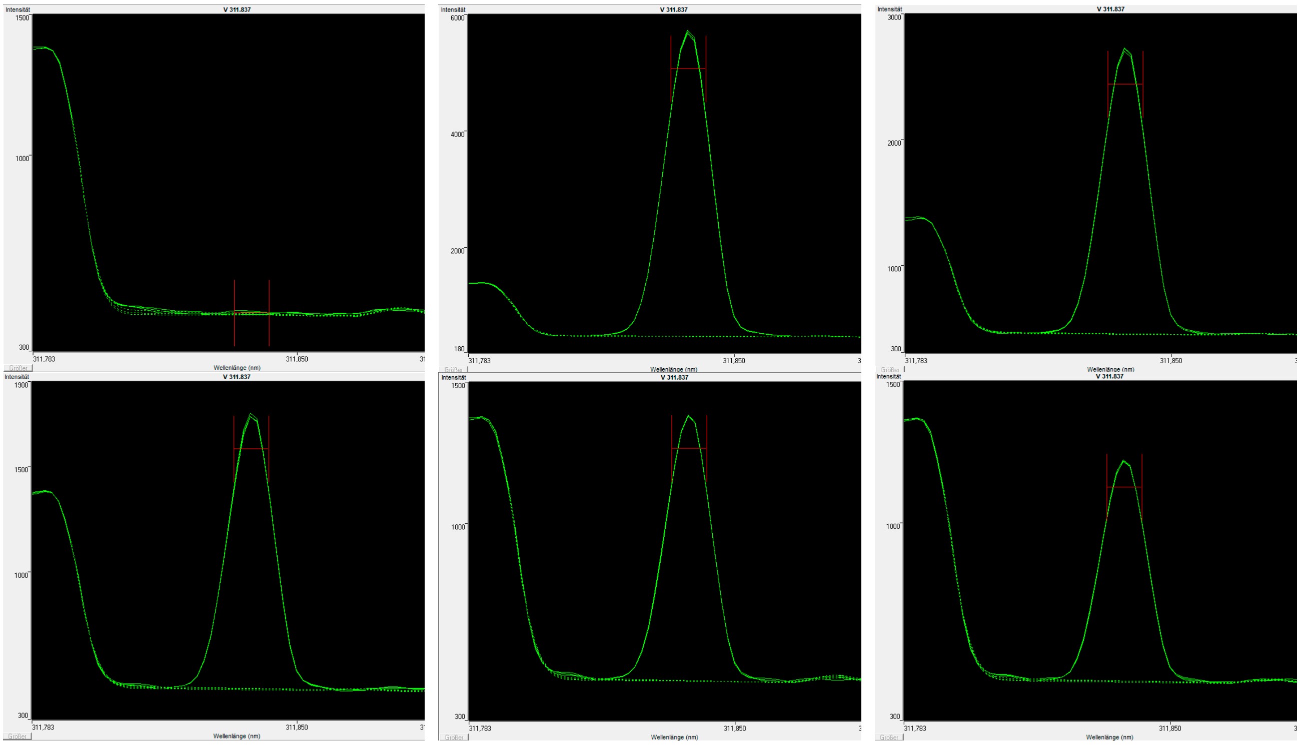

Figure S13. ICP-OES spectra of leached V (311.837 nm) in the reaction solutions of the catalytic set 3. Blank sample and reaction solutions from cycles 1-5 are presented from left to right.

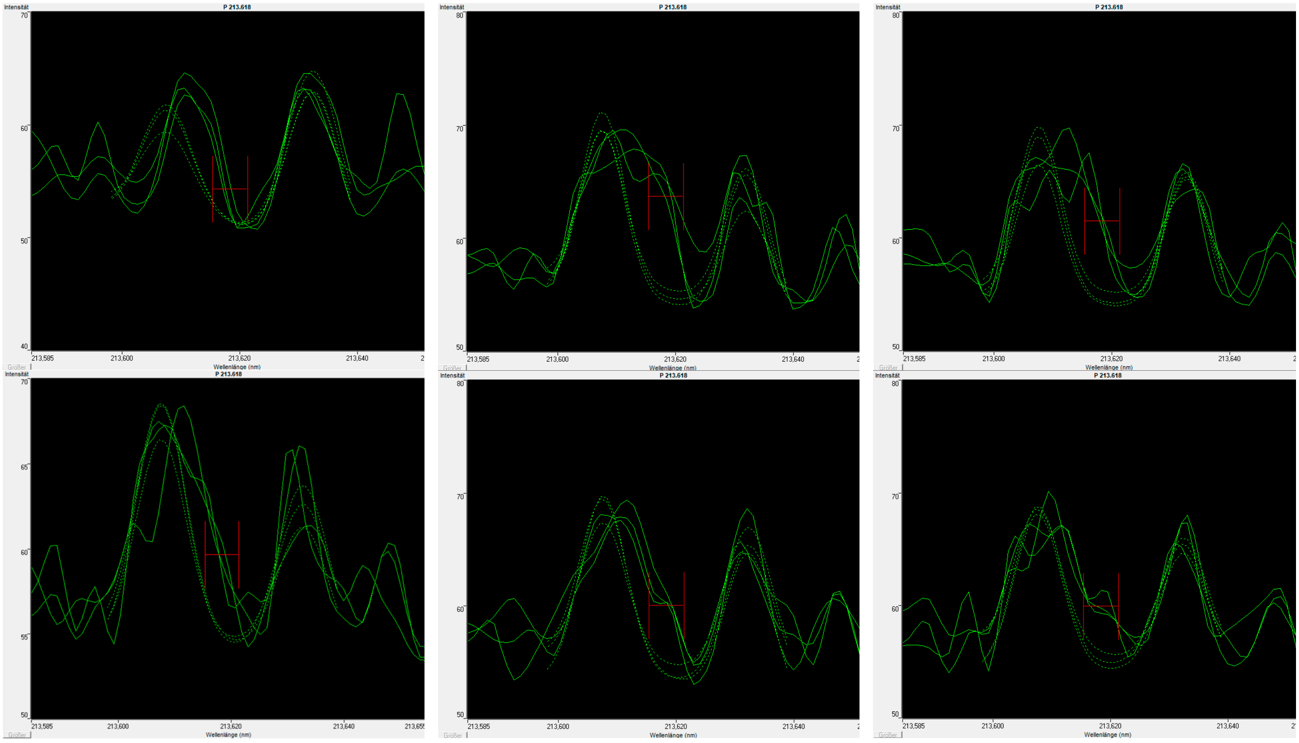

Figure S14. ICP-OES spectra of leached P (213.618 nm) in the reaction solutions of the catalytic set 2. Blank sample and reaction solutions from cycles 1-5 are presented from left to right.

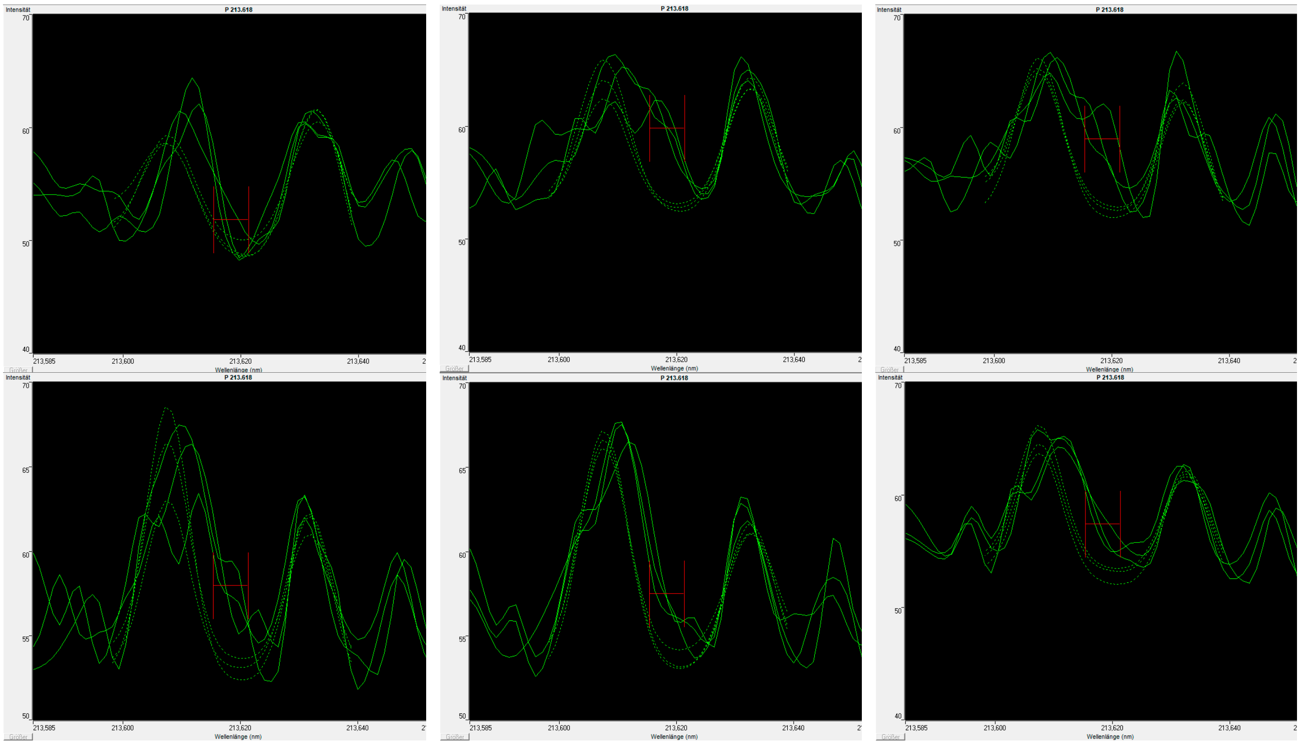

Figure S15. ICP-OES spectra of leached P (213.618 nm) in the reaction solutions of the catalytic set 3. Blank sample and reaction solutions from cycles 1-5 are presented from left to right.
